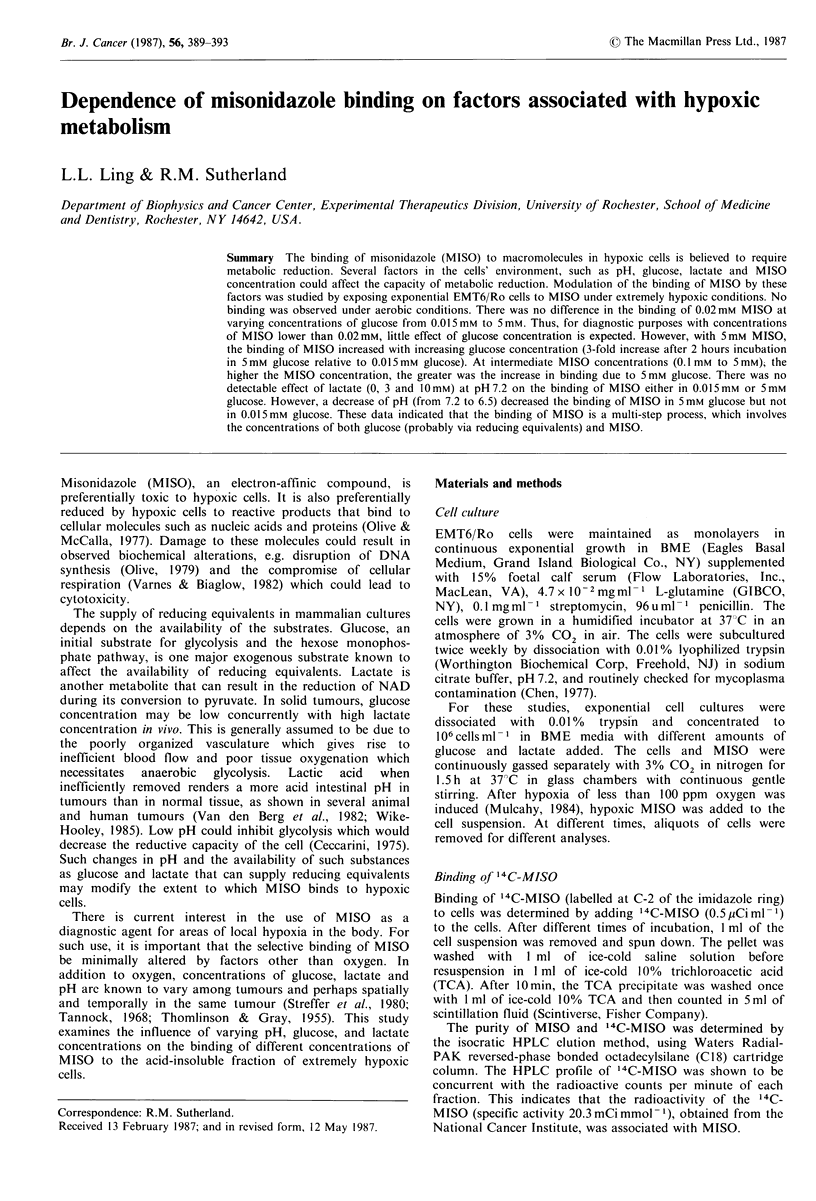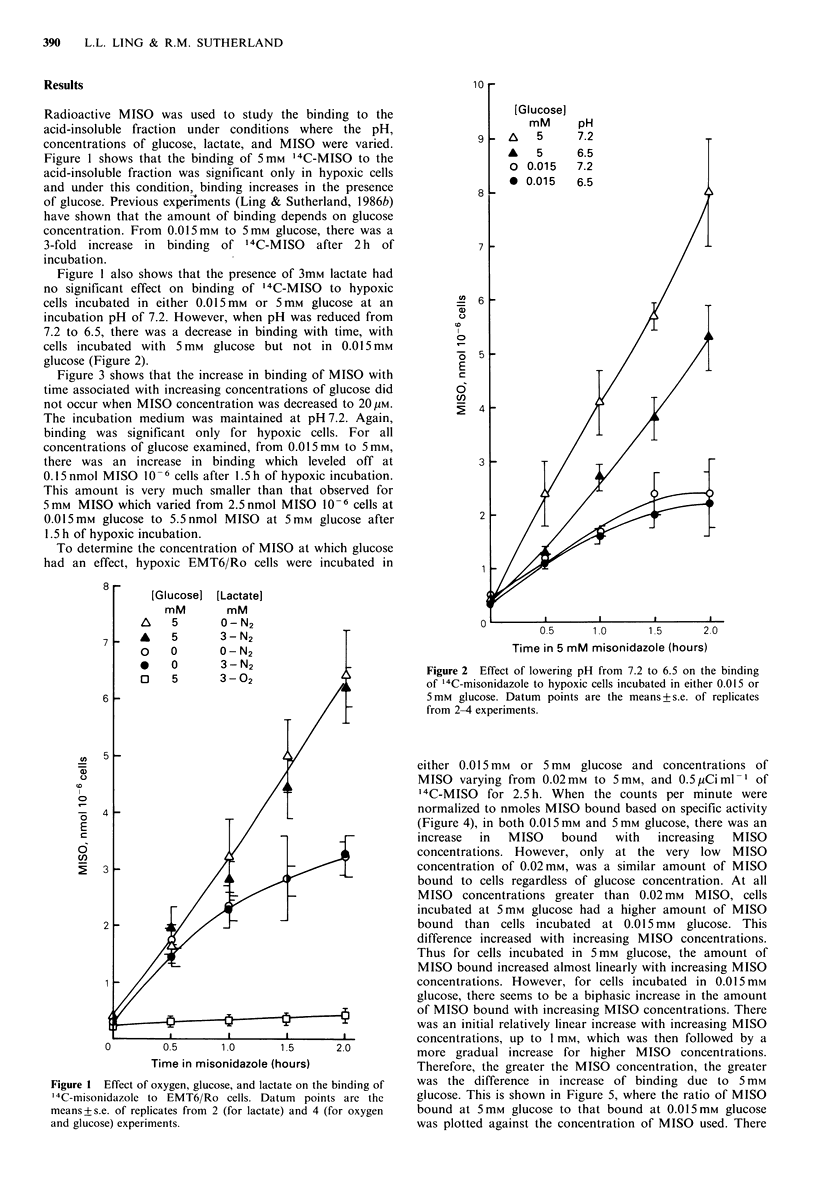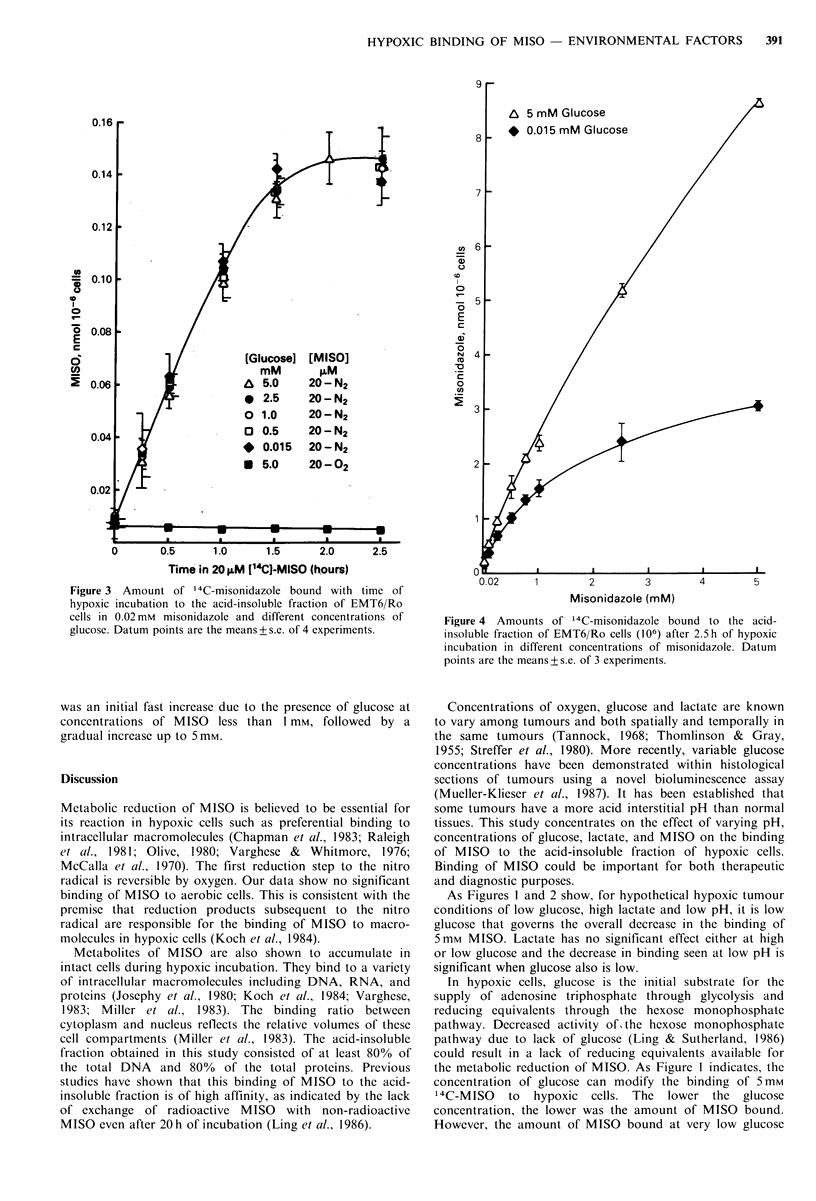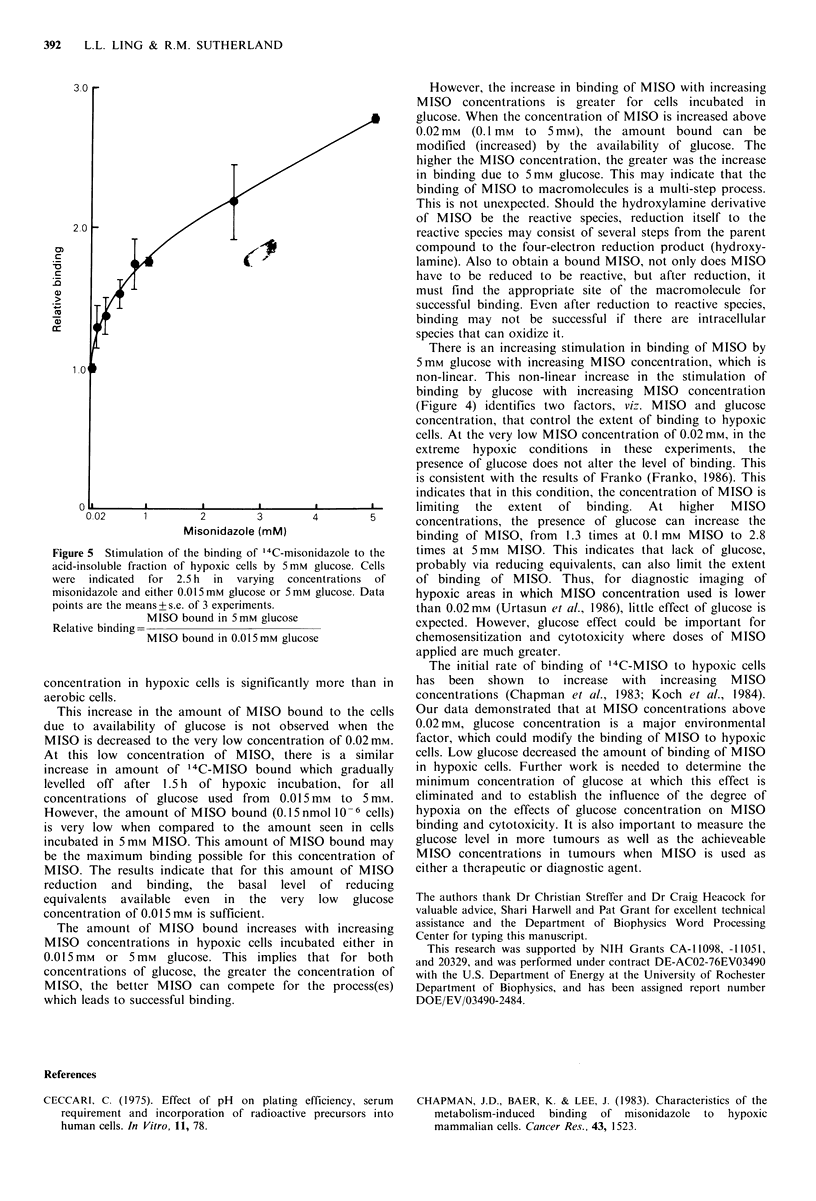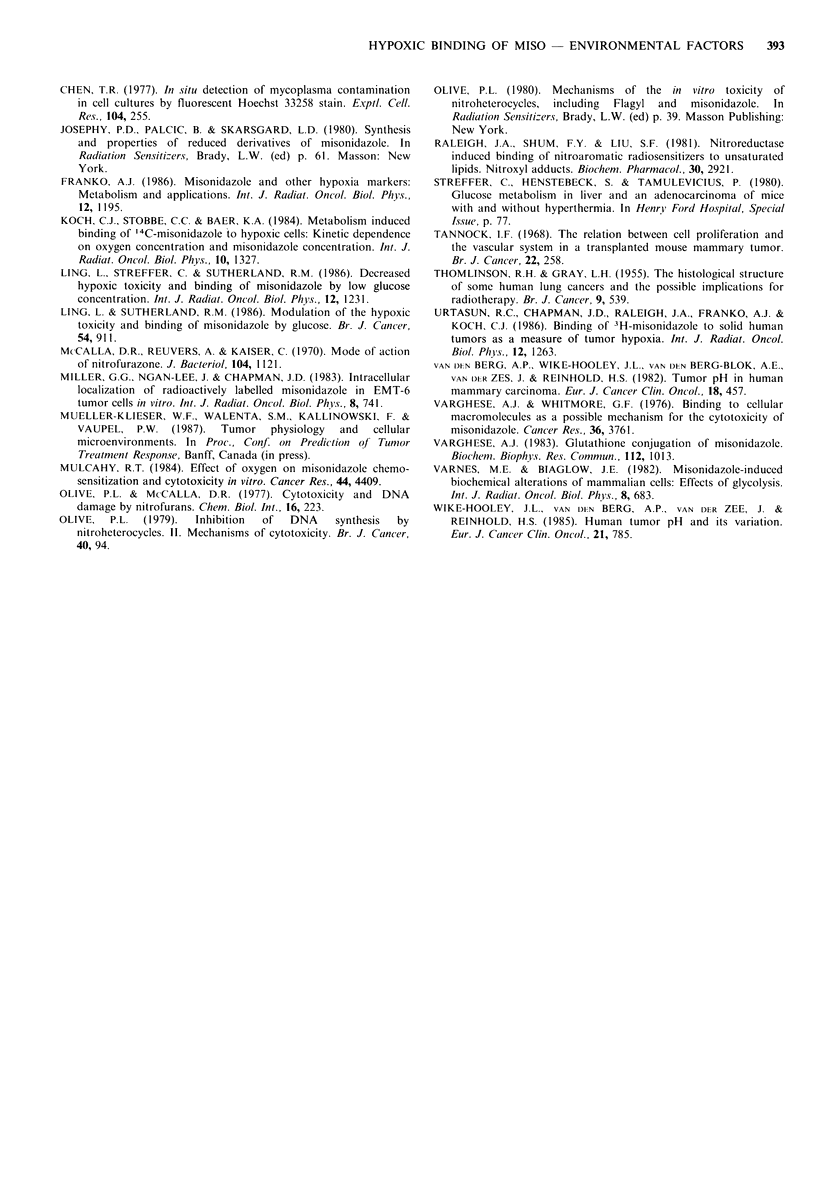# Dependence of misonidazole binding on factors associated with hypoxic metabolism.

**DOI:** 10.1038/bjc.1987.212

**Published:** 1987-10

**Authors:** L. L. Ling, R. M. Sutherland

**Affiliations:** Department of Biophysics and Cancer Center, University of Rochester, School of Medicine and Dentistry 14642.

## Abstract

The binding of misonidazole (MISO) to macromolecules in hypoxic cells is believed to require metabolic reduction. Several factors in the cells' environment, such as pH, glucose, lactate and MISO concentration could affect the capacity of metabolic reduction. Modulation of the binding of MISO by these factors was studied by exposing exponential EMT6/Ro cells to MISO under extremely hypoxic conditions. No binding was observed under aerobic conditions. There was no difference in the binding of 0.02 mM MISO at varying concentrations of glucose from 0.015 mM to 5 mM. Thus, for diagnostic purposes with concentrations of MISO lower than 0.02 mM, little effect of glucose concentration is expected. However, with 5 mM MISO, the binding of MISO increased with increasing glucose concentration (3-fold increase after 2 hours incubation in 5 mM glucose relative to 0.015 mM glucose). At intermediate MISO concentrations (0.1 mM to 5 mM); the higher the MISO concentration, the greater was the increase in binding due to 5 mM glucose. There was no detectable effect of lactate (0, 3 and 10 mM) at pH 7.2 on the binding of MISO either in 0.015 mM or 5 mM glucose. However, a decrease of pH (from 7.2 to 6.5) decreased the binding of MISO in 5 mM glucose but not in 0.015 mM glucose. These data indicated that the binding of MISO is a multi-step process, which involves the concentrations of both glucose (probably via reducing equivalents) and MISO.


*Br. J. Cancer* (1987), **56**, 389–393 © The Macmillan Press Ltd., 1987

### Dependence of misonidazole binding on factors associated with hypoxic metabolism

L.L. Ling & R.M. Sutherland

*Department of Biophysics and Cancer Center, Experimental Therapeutics Division, University of Rochester, School of Medicine and Dentistry, Rochester, NY 14642, USA.*

**Summary** The binding of misonidazole (MISO) to macromolecules in hypoxic cells is believed to require metabolic reduction. Several factors in the cells' environment, such as pH, glucose, lactate and MISO concentration could affect the capacity of metabolic reduction. Modulation of the binding of MISO by these factors was studied by exposing exponential EMT6/Ro cells to MISO under extremely hypoxic conditions. No binding was observed under aerobic conditions. There was no difference in the binding of 0.02 mM MISO at varying concentrations of glucose from 0.015 mM to 5 mM. Thus, for diagnostic purposes with concentrations of MISO lower than 0.02 mM, little effect of glucose concentration is expected. However, with 5 mM MISO, the binding of MISO increased with increasing glucose concentration (3-fold increase after 2 hours incubation in 5 mM glucose relative to 0.015 mM glucose). At intermediate MISO concentrations (0.1 mM to 5 mM); the higher the MISO concentration, the greater was the increase in binding due to 5 mM glucose. There was no detectable effect of lactate (0, 3 and 10 mM) at pH 7.2 on the binding of MISO either in 0.015 mM or 5 mM glucose. However, a decrease of pH (from 7.2 to 6.5) decreased the binding of MISO in 5 mM glucose but not in 0.015 mM glucose. These data indicated that the binding of MISO is a multi-step process, which involves the concentrations of both glucose (probably via reducing equivalents) and MISO.

Misonidazole (MISO), an electron-affinic compound, is preferentially toxic to hypoxic cells. It is also preferentially reduced by hypoxic cells to reactive products that bind to cellular molecules such as nucleic acids and proteins (Olive & McCalla, 1977). Damage to these molecules could result in observed biochemical alterations, e.g. disruption of DNA synthesis (Olive, 1979) and the compromise of cellular respiration (Varnes & Biaglow, 1982) which could lead to cytotoxicity.

The supply of reducing equivalents in mammalian cultures depends on the availability of the substrates. Glucose, an initial substrate for glycolysis and the hexose monophosphate pathway, is one major exogenous substrate known to affect the availability of reducing equivalents. Lactate is another metabolite that can result in the reduction of NAD during its conversion to pyruvate. In solid tumours, glucose concentration may be low concurrently with high lactate concentration *in vivo*. This is generally assumed to be due to the poorly organized vasculature which gives rise to inefficient blood flow and poor tissue oxygenation which necessitates anaerobic glycolysis. Lactic acid when inefficiently removed renders a more acid intestinal pH in tumours than in normal tissue, as shown in several animal and human tumours (Van den Berg *et al.*, 1982; Wike-Hooley, 1985). Low pH could inhibit glycolysis which would decrease the reductive capacity of the cell (Ceccarini, 1975). Such changes in pH and the availability of such substances as glucose and lactate that can supply reducing equivalents may modify the extent to which MISO binds to hypoxic cells.

There is current interest in the use of MISO as a diagnostic agent for areas of local hypoxia in the body. For such use, it is important that the selective binding of MISO be minimally altered by factors other than oxygen. In addition to oxygen, concentrations of glucose, lactate and pH are known to vary among tumours and perhaps spatially and temporally in the same tumour (Streffer *et al.*, 1980; Tannock, 1968; Thomlinson & Gray, 1955). This study examines the influence of varying pH, glucose, and lactate concentrations on the binding of different concentrations of MISO to the acid-insoluble fraction of extremely hypoxic cells.

Correspondence: R.M. Sutherland.
Received 13 February 1987; and in revised form, 12 May 1987.

### Materials and methods

### *Cell culture*

EMT6/Ro cells were maintained as monolayers in continuous exponential growth in BME (Eagles Basal Medium, Grand Island Biological Co., NY) supplemented with 15% foetal calf serum (Flow Laboratories, Inc., MacLean, VA), $4.7\times10^{-2}$ mg ml$^{-1}$ L-glutamine (GIBCO, NY), 0.1 mg ml$^{-1}$ streptomycin, 96 u ml$^{-1}$ penicillin. The cells were grown in a humidified incubator at 37°C in an atmosphere of 3% $CO_2$ in air. The cells were subcultured twice weekly by dissociation with 0.01% lyophilized trypsin (Worthington Biochemical Corp, Freehold, NJ) in sodium citrate buffer, pH 7.2, and routinely checked for mycoplasma contamination (Chen, 1977).

For these studies, exponential cell cultures were dissociated with 0.01% trypsin and concentrated to $10^6$ cells ml$^{-1}$ in BME media with different amounts of glucose and lactate added. The cells and MISO were continuously gassed separately with 3% $CO_2$ in nitrogen for 1.5 h at 37°C in glass chambers with continuous gentle stirring. After hypoxia of less than 100 ppm oxygen was induced (Mulcahy, 1984), hypoxic MISO was added to the cell suspension. At different times, aliquots of cells were removed for different analyses.

### *Binding of $^{14}$C-MISO*

Binding of $^{14}$C-MISO (labelled at C-2 of the imidazole ring) to cells was determined by adding $^{14}$C-MISO (0.5 $\mu$Ci ml$^{-1}$) to the cells. After different times of incubation, 1 ml of the cell suspension was removed and spun down. The pellet was washed with 1 ml of ice-cold saline solution before resuspension in 1 ml of ice-cold 10% trichloroacetic acid (TCA). After 10 min, the TCA precipitate was washed once with 1 ml of ice-cold 10% TCA and then counted in 5 ml of scintillation fluid (Scintiverse, Fisher Company).

The purity of MISO and $^{14}$C-MISO was determined by the isocratic HPLC elution method, using Waters Radial-PAK reversed-phase bonded octadecylsilane (C18) cartridge column. The HPLC profile of $^{14}$C-MISO was shown to be concurrent with the radioactive counts per minute of each fraction. This indicates that the radioactivity of the $^{14}$C-MISO (specific activity 20.3 mCi mmol$^{-1}$), obtained from the National Cancer Institute, was associated with MISO.

### Results

Radioactive MISO was used to study the binding to the acid-insoluble fraction under conditions where the pH, concentrations of glucose, lactate, and MISO were varied. Figure 1 shows that the binding of 5 mM $^{14}$C-MISO to the acid-insoluble fraction was significant only in hypoxic cells and under this condition, binding increases in the presence of glucose. Previous experiments (Ling & Sutherland, 1986*b*) have shown that the amount of binding depends on glucose concentration. From 0.015 mM to 5 mM glucose, there was a 3-fold increase in binding of $^{14}$C-MISO after 2 h of incubation.

Figure 1 also shows that the presence of 3 mM lactate had no significant effect on binding of $^{14}$C-MISO to hypoxic cells incubated in either 0.015 mM or 5 mM glucose at an incubation pH of 7.2. However, when pH was reduced from 7.2 to 6.5, there was a decrease in binding with time, with cells incubated with 5 mM glucose but not in 0.015 mM glucose (Figure 2).

Figure 3 shows that the increase in binding of MISO with time associated with increasing concentrations of glucose did not occur when MISO concentration was decreased to 20 $\mu$M. The incubation medium was maintained at pH 7.2. Again, binding was significant only for hypoxic cells. For all concentrations of glucose examined, from 0.015 mM to 5 mM, there was an increase in binding which leveled off at 0.15 nmol MISO $10^{-6}$ cells after 1.5 h of hypoxic incubation. This amount is very much smaller than that observed for 5 mM MISO which varied from 2.5 nmol MISO $10^{-6}$ cells at 0.015 mM glucose to 5.5 nmol MISO at 5 mM glucose after 1.5 h of hypoxic incubation.

To determine the concentration of MISO at which glucose had an effect, hypoxic EMT6/Ro cells were incubated in either 0.015 mM or 5 mM glucose and concentrations of MISO varying from 0.02 mM to 5 mM, and 0.5 $\mu$Ci ml$^{-1}$ of $^{14}$C-MISO for 2.5 h. When the counts per minute were normalized to nmoles MISO bound based on specific activity (Figure 4), in both 0.015 mM and 5 mM glucose, there was an increase in MISO bound with increasing MISO concentrations. However, only at the very low MISO concentration of 0.02 mM, was a similar amount of MISO bound to cells regardless of glucose concentration. At all MISO concentrations greater than 0.02 mM MISO, cells incubated at 5 mM glucose had a higher amount of MISO bound than cells incubated at 0.015 mM glucose. This difference increased with increasing MISO concentrations. Thus for cells incubated in 5 mM glucose, the amount of MISO bound increased almost linearly with increasing MISO concentrations. However, for cells incubated in 0.015 mM glucose, there seems to be a biphasic increase in the amount of MISO bound with increasing MISO concentrations. There was an initial relatively linear increase with increasing MISO concentrations, up to 1 mM, which was then followed by a more gradual increase for higher MISO concentrations. Therefore, the greater the MISO concentration, the greater was the difference in increase of binding due to 5 mM glucose. This is shown in Figure 5, where the ratio of MISO bound at 5 mM glucose to that bound at 0.015 mM glucose was plotted against the concentration of MISO used. There

**Figure 1** Effect of oxygen, glucose, and lactate on the binding of $^{14}$C-misonidazole to EMT6/Ro cells. Datum points are the means ± s.e. of replicates from 2 (for lactate) and 4 (for oxygen and glucose) experiments.

**Figure 2** Effect of lowering pH from 7.2 to 6.5 on the binding of $^{14}$C-misonidazole to hypoxic cells incubated in either 0.015 or 5 mM glucose. Datum points are the means ± s.e. of replicates from 2–4 experiments.

**Figure 3** Amount of $^{14}$C-misonidazole bound with time of hypoxic incubation to the acid-insoluble fraction of EMT6/Ro cells in 0.02 mM misonidazole and different concentrations of glucose. Datum points are the means ± s.e. of 4 experiments.

**Figure 4** Amounts of $^{14}$C-misonidazole bound to the acid-insoluble fraction of EMT6/Ro cells ($10^6$) after 2.5 h of hypoxic incubation in different concentrations of misonidazole. Datum points are the means ± s.e. of 3 experiments.

was an initial fast increase due to the presence of glucose at concentrations of MISO less than 1 mM, followed by a gradual increase up to 5 mM.

### Discussion

Metabolic reduction of MISO is believed to be essential for its reaction in hypoxic cells such as preferential binding to intracellular macromolecules (Chapman *et al.*, 1983; Raleigh *et al.*, 1981; Olive, 1980; Varghese & Whitmore, 1976; McCalla *et al.*, 1970). The first reduction step to the nitro radical is reversible by oxygen. Our data show no significant binding of MISO to aerobic cells. This is consistent with the premise that reduction products subsequent to the nitro radical are responsible for the binding of MISO to macromolecules in hypoxic cells (Koch *et al.*, 1984).

Metabolites of MISO are also shown to accumulate in intact cells during hypoxic incubation. They bind to a variety of intracellular macromolecules including DNA, RNA, and proteins (Josephy *et al.*, 1980; Koch *et al.*, 1984; Varghese, 1983; Miller *et al.*, 1983). The binding ratio between cytoplasm and nucleus reflects the relative volumes of these cell compartments (Miller *et al.*, 1983). The acid-insoluble fraction obtained in this study consisted of at least 80% of the total DNA and 80% of the total proteins. Previous studies have shown that this binding of MISO to the acid-insoluble fraction is of high affinity, as indicated by the lack of exchange of radioactive MISO with non-radioactive MISO even after 20 h of incubation (Ling *et al.*, 1986).

Concentrations of oxygen, glucose and lactate are known to vary among tumours and both spatially and temporally in the same tumours (Tannock, 1968; Thomlinson & Gray, 1955; Streffer *et al.*, 1980). More recently, variable glucose concentrations have been demonstrated within histological sections of tumours using a novel bioluminescence assay (Mueller-Klieser *et al.*, 1987). It has been established that some tumours have a more acid interstitial pH than normal tissues. This study concentrates on the effect of varying pH, concentrations of glucose, lactate, and MISO on the binding of MISO to the acid-insoluble fraction of hypoxic cells. Binding of MISO could be important for both therapeutic and diagnostic purposes.

As Figures 1 and 2 show, for hypothetical hypoxic tumour conditions of low glucose, high lactate and low pH, it is low glucose that governs the overall decrease in the binding of 5 mM MISO. Lactate has no significant effect either at high or low glucose and the decrease in binding seen at low pH is significant when glucose also is low.

In hypoxic cells, glucose is the initial substrate for the supply of adenosine triphosphate through glycolysis and reducing equivalents through the hexose monophosphate pathway. Decreased activity of the hexose monophosphate pathway due to lack of glucose (Ling & Sutherland, 1986) could result in a lack of reducing equivalents available for the metabolic reduction of MISO. As Figure 1 indicates, the concentration of glucose can modify the binding of 5 mM $^{14}$C-MISO to hypoxic cells. The lower the glucose concentration, the lower was the amount of MISO bound. However, the amount of MISO bound at very low glucose

**Figure 5** Stimulation of the binding of $^{14}$C-misonidazole to the acid-insoluble fraction of hypoxic cells by 5 mM glucose. Cells were indicated for 2.5 h in varying concentrations of misonidazole and either 0.015 mM glucose or 5 mM glucose. Data points are the means ± s.e. of 3 experiments.

$$\text{Relative binding} = \frac{\text{MISO bound in 5 mM glucose}}{\text{MISO bound in 0.015 mM glucose}}$$

concentration in hypoxic cells is significantly more than in aerobic cells.

This increase in the amount of MISO bound to the cells due to availability of glucose is not observed when the MISO is decreased to the very low concentration of 0.02 mM. At this low concentration of MISO, there is a similar increase in amount of $^{14}$C-MISO bound which gradually levelled off after 1.5 h of hypoxic incubation, for all concentrations of glucose used from 0.015 mM to 5 mM. However, the amount of MISO bound (0.15 nmol $10^{-6}$ cells) is very low when compared to the amount seen in cells incubated in 5 mM MISO. This amount of MISO bound may be the maximum binding possible for this concentration of MISO. The results indicate that for this amount of MISO reduction and binding, the basal level of reducing equivalents available even in the very low glucose concentration of 0.015 mM is sufficient.

The amount of MISO bound increases with increasing MISO concentrations in hypoxic cells incubated either in 0.015 mM or 5 mM glucose. This implies that for both concentrations of glucose, the greater the concentration of MISO, the better MISO can compete for the process(es) which leads to successful binding.

However, the increase in binding of MISO with increasing MISO concentrations is greater for cells incubated in glucose. When the concentration of MISO is increased above 0.02 mM (0.1 mM to 5 mM), the amount bound can be modified (increased) by the availability of glucose. The higher the MISO concentration, the greater was the increase in binding due to 5 mM glucose. This may indicate that the binding of MISO to macromolecules is a multi-step process. This is not unexpected. Should the hydroxylamine derivative of MISO be the reactive species, reduction itself to the reactive species may consist of several steps from the parent compound to the four-electron reduction product (hydroxylamine). Also to obtain a bound MISO, not only does MISO have to be reduced to be reactive, but after reduction, it must find the appropriate site of the macromolecule for successful binding. Even after reduction to reactive species, binding may not be successful if there are intracellular species that can oxidize it.

There is an increasing stimulation in binding of MISO by 5 mM glucose with increasing MISO concentration, which is non-linear. This non-linear increase in the stimulation of binding by glucose with increasing MISO concentration (Figure 4) identifies two factors, *viz.* MISO and glucose concentration, that control the extent of binding to hypoxic cells. At the very low MISO concentration of 0.02 mM, in the extreme hypoxic conditions in these experiments, the presence of glucose does not alter the level of binding. This is consistent with the results of Franko (Franko, 1986). This indicates that in this condition, the concentration of MISO is limiting the extent of binding. At higher MISO concentrations, the presence of glucose can increase the binding of MISO, from 1.3 times at 0.1 mM MISO to 2.8 times at 5 mM MISO. This indicates that lack of glucose, probably via reducing equivalents, can also limit the extent of binding of MISO. Thus, for diagnostic imaging of hypoxic areas in which MISO concentration used is lower than 0.02 mM (Urtasun *et al.*, 1986), little effect of glucose is expected. However, glucose effect could be important for chemosensitization and cytotoxicity where doses of MISO applied are much greater.

The initial rate of binding of $^{14}$C-MISO to hypoxic cells has been shown to increase with increasing MISO concentrations (Chapman *et al.*, 1983; Koch *et al.*, 1984). Our data demonstrated that at MISO concentrations above 0.02 mM, glucose concentration is a major environmental factor, which could modify the binding of MISO to hypoxic cells. Low glucose decreased the amount of binding of MISO in hypoxic cells. Further work is needed to determine the minimum concentration of glucose at which this effect is eliminated and to establish the influence of the degree of hypoxia on the effects of glucose concentration on MISO binding and cytotoxicity. It is also important to measure the glucose level in more tumours as well as the achieveable MISO concentrations in tumours when MISO is used as either a therapeutic or diagnostic agent.

The authors thank Dr Christian Streffer and Dr Craig Heacock for valuable advice, Shari Harwell and Pat Grant for excellent technical assistance and the Department of Biophysics Word Processing Center for typing this manuscript.

This research was supported by NIH Grants CA-11098, -11051, and 20329, and was performed under contract DE-AC02-76EV03490 with the U.S. Department of Energy at the University of Rochester Department of Biophysics, and has been assigned report number DOE/EV/03490-2484.

### References

CECCARI, C. (1975). Effect of pH on plating efficiency, serum requirement and incorporation of radioactive precursors into human cells. *In Vitro*, **11**, 78.

CHAPMAN, J.D., BAER, K. & LEE, J. (1983). Characteristics of the metabolism-induced binding of misonidazole to hypoxic mammalian cells. *Cancer Res.*, **43**, 1523.

CHEN, T.R. (1977). *In situ* detection of mycoplasma contamination in cell cultures by fluorescent Hoechst 33258 stain. *Exptl. Cell. Res.*, **104,** 255.

JOSEPHY, P.D., PALCIC, B. & SKARSGARD, L.D. (1980). Synthesis and properties of reduced derivatives of misonidazole. In *Radiation Sensitizers*, Brady, L.W. (ed) p. 61. Masson: New York.

FRANKO, A.J. (1986). Misonidazole and other hypoxia markers: Metabolism and applications. *Int. J. Radiat. Oncol. Biol. Phys.*, **12,** 1195.

KOCH, C.J., STOBBE, C.C. & BAER, K.A. (1984). Metabolism induced binding of $^{14}C$-misonidazole to hypoxic cells: Kinetic dependence on oxygen concentration and misonidazole concentration. *Int. J. Radiat. Oncol. Biol. Phys.*, **10,** 1327.

LING, L., STREFFER, C. & SUTHERLAND, R.M. (1986). Decreased hypoxic toxicity and binding of misonidazole by low glucose concentration. *Int. J. Radiat. Oncol. Biol. Phys.*, **12,** 1231.

LING, L. & SUTHERLAND, R.M. (1986). Modulation of the hypoxic toxicity and binding of misonidazole by glucose. *Br. J. Cancer*, **54,** 911.

McCALLA, D.R., REUVERS, A. & KAISER, C. (1970). Mode of action of nitrofurazone. *J. Bacteriol*, **104,** 1121.

MILLER, G.G., NGAN-LEE, J. & CHAPMAN, J.D. (1983). Intracellular localization of radioactively labelled misonidazole in EMT-6 tumor cells *in vitro*. *Int. J. Radiat. Oncol. Biol. Phys.*, **8,** 741.

MUELLER-KLIESER, W.F., WALENTA, S.M., KALLINOWSKI, F. & VAUPEL, P.W. (1987). Tumor physiology and cellular microenvironments. In *Proc., Conf. on Prediction of Tumor Treatment Response*, Banff, Canada (in press).

MULCAHY, R.T. (1984). Effect of oxygen on misonidazole chemo-sensitization and cytotoxicity *in vitro*. *Cancer Res.*, **44,** 4409.

OLIVE, P.L. & McCALLA, D.R. (1977). Cytotoxicity and DNA damage by nitrofurans. *Chem. Biol. Int.*, **16,** 223.

OLIVE, P.L. (1979). Inhibition of DNA synthesis by nitroheterocycles. II. Mechanisms of cytotoxicity. *Br. J. Cancer*, **40,** 94.

OLIVE, P.L. (1980). Mechanisms of the *in vitro* toxicity of nitroheterocycles, including Flagyl and misonidazole. In *Radiation Sensitizers*, Brady, L.W. (ed) p. 39. Masson Publishing: New York.

RALEIGH, J.A., SHUM, F.Y. & LIU, S.F. (1981). Nitroreductase induced binding of nitroaromatic radiosensitizers to unsaturated lipids. Nitroxyl adducts. *Biochem. Pharmacol.*, **30,** 2921.

STREFFER, C., HENSTEBECK, S. & TAMULEVICIUS, P. (1980). Glucose metabolism in liver and an adenocarcinoma of mice with and without hyperthermia. In *Henry Ford Hospital, Special Issue*, p. 77.

TANNOCK, I.F. (1968). The relation between cell proliferation and the vascular system in a transplanted mouse mammary tumor. *Br. J. Cancer*, **22,** 258.

THOMLINSON, R.H. & GRAY, L.H. (1955). The histological structure of some human lung cancers and the possible implications for radiotherapy. *Br. J. Cancer*, **9,** 539.

URTASUN, R.C., CHAPMAN, J.D., RALEIGH, J.A., FRANKO, A.J. & KOCH, C.J. (1986). Binding of $^{3}H$-misonidazole to solid human tumors as a measure of tumor hypoxia. *Int. J. Radiat. Oncol. Biol. Phys.*, **12,** 1263.

VAN DEN BERG, A.P., WIKE-HOOLEY, J.L., VAN DEN BERG-BLOK, A.E., VAN DER ZES, J. & REINHOLD, H.S. (1982). Tumor pH in human mammary carcinoma. *Eur. J. Cancer Clin. Oncol.*, **18,** 457.

VARGHESE, A.J. & WHITMORE, G.F. (1976). Binding to cellular macromolecules as a possible mechanism for the cytotoxicity of misonidazole. *Cancer Res.*, **36,** 3761.

VARGHESE, A.J. (1983). Glutathione conjugation of misonidazole. *Biochem. Biophys. Res. Commun.*, **112,** 1013.

VARNES, M.E. & BIAGLOW, J.E. (1982). Misonidazole-induced biochemical alterations of mammalian cells: Effects of glycolysis. *Int. J. Radiat. Oncol. Biol. Phys.*, **8,** 683.

WIKE-HOOLEY, J.L., VAN DEN BERG, A.P., VAN DER ZEE, J. & REINHOLD, H.S. (1985). Human tumor pH and its variation. *Eur. J. Cancer Clin. Oncol.*, **21,** 785.